# Modifiable Innate Biology within the Gut–Brain Axis for Alzheimer’s Disease

**DOI:** 10.3390/biomedicines10092098

**Published:** 2022-08-27

**Authors:** Helena Marcos Pasero, Aurora García Tejedor, Juan Antonio Giménez-Bastida, José Moisés Laparra Llopis

**Affiliations:** 1 Bioactivity and Nutritional Immunology Group (BIOINUT), Faculty of Health Sciences, Universidad Internacional de Valencia—VIU, Pintor Sorolla 21, 46002 Valencia, Spain; 2 Laboratory of Food and Health, Research Group on Quality, Safety and Bioactivity of Plant Foods, Department Food Science and Technology, CEBAS-CSIC, Campus de Espinardo, 30100 Murcia, Spain; 3 Molecular Immunonutrition Group, Madrid Institute for Advanced Studies in Food (IMDEA Food), Ctra Cantoblanco 8, 28049 Madrid, Spain

**Keywords:** Alzheimer’s disease, microglia, immunonutrition, innate immunity

## Abstract

Alzheimer’s disease (AD) is a prototypical inflammation-associated loss of cognitive function, with approximately 90% of the AD burden associated with invading myeloid cells controlling the function of the resident microglia. This indicates that the immune microenvironment has a pivotal role in the pathogenesis of the disease. Multiple peripheral stimuli, conditioned by complex and varied interactions between signals that stem at the intestinal level and neuroimmune processes, are involved in the progression and severity of AD. Conceivably, the targeting of critical innate immune signals and cells is achievable, influencing immune and metabolic health within the gut–brain axis. Considerable progress has been made, modulating many different metabolic and immune alterations that can drive AD development. However, non-pharmacological strategies targeting immunometabolic processes affecting neuroinflammation in AD treatment remain general and, at this point, are applied to all patients regardless of disease features. Despite these possibilities, improved knowledge of the relative contribution of the different innate immune cells and molecules comprising the chronically inflamed brain network to AD pathogenesis, and elucidation of the network hierarchy, are needed for planning potent preventive and/or therapeutic interventions. Moreover, an integrative perspective addressing transdisciplinary fields can significantly contribute to molecular pathological epidemiology, improving the health and quality of life of AD patients. This review is intended to gather modifiable immunometabolic processes based on their importance in the prevention and management of AD.

## 1. Introduction

Alzheimer’s disease (AD) has become one of the fastest-increasing diseases worldwide, accounting for 50% to 75% of all cases of dementia. AD is estimated to affect 6.5 million Americans aged 65 and older, while in 2006 patients in Europe were calculated to number up to 16.51 million [1]. Far from being controlled, recent studies predict negative forecasts for the disease, increasing by up to 87% in Europe for the 2010–2050 period [2].

The etiology of the neurodegeneration in AD is still unknown, although the prevailing hypothesis considers the deposition of amyloid-β (Aβ) plaques to be the initiating event. Here, immune imbalances causing an inability to eliminate Aβ accumulation lead to an immunosuppressive microenvironment favoring pathological disorders. These are accompanied by chronic inflammation that fails to resolve itself, where the production of anti-inflammatory cytokines contributes to increasing Aβ deposition. Microglia play major roles in neuroinflammation, which is associated with the development of different phenotypes under specific conditions. Recent genetic studies identified candidate genes (>40 loci) that are expressed in both microglia and myeloid cells, which infiltrate into the brain, as disease risks for AD [3]. Otherwise, various risk factors for AD are well-defined, including age, familial inheritance, exposure to aluminum, traumatic brain injury, and associated co-morbidities such as vascular disease and infection [4]. Only rare forms of early-onset familial AD display causal gene mutations (i.e., amyloid precursor protein and presenilin). Late-onset sporadic AD presents as a multifactorial disorder including age, genetic factors, vascular diseases, and traumatic brain injury; factors associated with diet, immunity and infections are all implicated.

Over the past decade, there has been an improvement in the understanding of the genetic pathogenesis of AD; however, products of genes strongly associated with late-onset AD do not appear to be druggable targets [5]. Pharmacological strategies to overcome pathological manifestations in AD display partial activity, retarding rather than resolving the disease [6]. Notably, recent data linked peripheral inflammation-associated conditions to metabolic derangements in AD, suggesting their significant contribution to the pathophysiology and clinical symptoms of the disease [7,8]. Thus, more effective and complementary intervention strategies displaying a preventive or therapeutic character could provide substantial contributions to control key immune and metabolic imbalances aggravating the disease. An improved knowledge of the contribution of environmental factors (i.e., diet, nutrients, microbiota) to central nervous system (CNS) physiology could constitute the basis of these strategies.

Environmental factors are addressable via preventive or therapeutic intentions influencing common immunometabolic imbalances, such as insulin production/resistance, as well as lipid homeostasis. In this sense, recent discoveries have established complex and varied interactions between signals that stem from the intestinal level, the severity of neuroinflammatory processes, and preservation of the cognitive function [9]. Strategies to influence brain inflammation, microglia and astroglia activation and disruption of the blood–brain barrier (BBB) are actively being investigated [10,11]. From a non-pharmacological point of view, the intestinal hypothesis for ‘inflammaging’ is based on the senescence of gut-associated lymphoid tissue (GALT) functions, and intestinal dysbiosis affecting intestinal integrity and barrier function [10]. This approach takes into consideration different players involved in the development and maintenance of brain homeostasis. However, the approach remains fragmented without the targeting of major immune signals and mediators (i.e., TLR4 signaling, monocyte-derived macrophages activation) that could interact with the mechanism triggered by the pathology of AD.

Previous research characterized plant seeds as a good source of proteins that display innate immune and metabolic effects triggered at the intestinal level [12]. For example, bioactive serine-type protease inhibitors (SETIs) appear to be mainly responsible for innate immune signals showing correlation with the stimulation of the innate immune Toll-like receptor (TLR)-4 signaling [13]. The role of TLR4 in AD remains ill-defined; for example, TLR4 mutation reduces microglial activation but increases Aβ deposits [14], while its role in the early stage of AD is unknown. SETI administration enables better control of lipid homeostasis, which is reflected in normalized variations of the hepatic pattern of major lipids (i.e., saturated fatty acids, cholesterol-associated products, MUFA, PUFA) [12,15]. This could have important consequences in vivo due to the preclinical demonstration of microglial activation in response to a high fat diet (HFD) or palmitate in an age-independent manner [16]. In addition, increased gene expression of markers such as CX3CR1, MHC-II, and NLRP3 was found, which could imply the participation of additional mediators in the exaggerated inflammatory response observed. When considering the immunometabolic context for AD development, it also has to be taken into account that Aβ is secreted as an apolipoprotein in nascent triglyceride-rich lipoproteins derived from both the liver and the intestine [17]. Thus, it could be hypothesized that environmental modulation of innate immune responses within the gut–liver axis could help modulate AD. The chemokine receptor CX3CR1 is expressed in various cells of the macrophage lineage. Moreover, emerging data highlight the beneficial potential of the CX3CL1–CX3CR1 axis in the pathogenesis of AD [18]. Feeding SETIs to animals under a HFD with chemically injured livers resulted in increased proportions of hepatic CD68^+^CX3CR1^+^CD74^+^ cells [19]. The potential role of CD74 in interacting with amyloid precursor protein (APP), inhibiting production, is also known [20]. Thus, as a consequence of intestinal environmental innate immune stimulatory conditions, subsets of monocyte-derived cells can generate a broad and diverse spectrum of immunometabolic responses, which may contribute to improvement in AD development and severity. The latter examples allow us to speculate regarding beneficial effects of immunometabolic strategies for AD patients.

In view of the pivotal role that the intestinal and, consequently, peripheral inflammatory microenvironments play in the natural history of AD, this review discusses potential immunomodulatory mechanisms of naturally occurring compounds that could counter a microenvironment favoring AD development, and their current reflection in intervention strategies. As such, this article is intended to gather immunometabolic processes based on their importance in the prevention and management of AD.

## 2. Targeting Innate Immunity in Alzheimer’s Disease

### 2.1. Gut–Liver–Brain Axis

**Figure 1:**
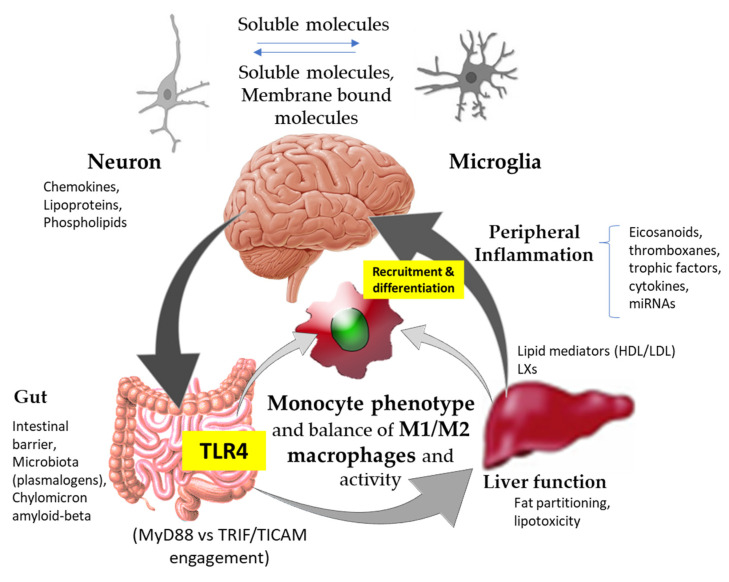
Schematic representation of physiological and biochemical pathways to approach the modifiable factors influencing innate immunity within the gut–liver–brain axis for Alzheimer’s disease prevention and/or treatment. TLR4, innate immune Toll-like receptor (TLR)-4; HDL, high-density lipoprotein; LDL, low-density lipoprotein; LXs, lipoxygenase interaction products.

A critical review of the scientific literature highlights the significant impact that signals stemming from different anatomical locations exert, influencing innate immune activation and peripheral inflammation (Figure 1). Neuroinflammation-mediated Aβ accumulation affects innate immunity, hampering its activity to rebalance central nervous system (CNS) biology. To this end, in recent years, several strategies have been investigated for the treatment of AD, including: (i) dampening of neuroinflammation, (ii) inhibition of key anti-inflammatory cytokines [21], and (iii) development of small molecules able to act at the peripheral immunity level (i.e., TLR4, macrophage colony stimulating factor, and NOD2 receptors) [6]. The latter represent a step forward from the classical approach to neuroinflammation towards an active innate immune targeted therapy. Here, peripheral inflammation was also revealed as a new player playing important roles in the innate immune function and activity of microglia [9,22,23].

Microglial cells are key players in immune response(s) within the CNS as first line of defense, not only to pathogens (i.e., pathogen-associated molecular patterns—PAMPs) but also to molecules and products released by damaged cells (i.e., damage-associated molecular patterns—DAMPs). Recognition of both PAMPs and DAMPs takes place via innate immune receptors such as Toll-like receptors (TLRs), nucleotide oligomerization domain (NOD)-like receptors, and C-type lectin receptors [24]. The capacity to coordinate the elimination of these stimuli, preventing aberrant activation of inflammatory responses, is essential to avoiding impairments in these mechanisms and maintaining adequate physiological response(s) in the CNS. Here, microglia play a dual role, either improving or worsening the disease: (i) a protective effect, by mediating amyloid beta (Aβ) clearance and degradation [25], and (ii) a negative impact favoring the spread of disease, by contributing to the propagation [26] and formation of neurotoxic forms [27] of Aβ plaques. Inflammatory responses influence the activation status of microglia and subsequently regulate their ability to take up and degrade Aβ, an area where ApoE and its receptors have been shown to play critical roles.

### 2.2. Innate Immune Receptors

Emerging evidence has established complex and varied interactions between signals that stem from the intestinal level and the severity of neuroinflammatory processes and preservation of cognitive function. Recent research highlighted the significant role that pharmacological small molecules tackling peripheral immunity can play in AD [21]. Similarly, preclinical studies revealed that different mechanisms initiated by extracts of plants (i.e., acetylcholinesterase (AChE) inhibition, increase of monoamines, anti-amyloid aggregation effect, and antioxidant activity) suppressed Aβ accumulation in mice [28,29].

The importance of peripheral inflammation is recognized because of its direct link to neuroinflammatory processes, as well as to the enhancement of monocyte infiltration and trafficking to regulate brain homeostasis [22,23]. For example, microglia as macrophages and monocytes express, among others, the triggering receptor expressed on myeloid cells-2 (TREM2), which is closely associated with AD [30]. TREM2-dependent signaling and cellular function is interfered with by the inhibition of γ-secretase activity, thereby decreasing phagocytic activity in macrophages and monocytes [31]. Impairments of these mechanisms favor the development of aberrations in immune and inflammatory processes of the disease, due to abnormal degradation and/or clearance. TREM2 exerts negative regulatory action on TLR4, among others, preventing astrocyte polarization into a proinflammatory phenotype and induction of neurodegenerative processes [32]. TLR4 knockout mice display higher expression levels of TREM2, which is associated with ameliorated neuroinflammation and improved neurological function. Hyperactivation of monocytes and macrophages in response to TLR2 and TLR4 stimulation in ‘mild cognitive impairment’ patients contributes to the progression of Alzheimer’s disease [33]. Although TLR4 appears to play a major role in AD, preclinical studies inhibiting TLR2 activation have also shown this receptor to be effective in attenuating Aβ accumulation and glial activation.

The impact of innate immune signals on intestinal and hepatic metabolic control, which directly affects peripheral inflammation, suggests that a multi-anatomical perspective should be considered to counter the negative manifestations of AD in the CNS (Figure 1).

## 3. A Journey between Modifiable Factors within the Gut–Liver–Brain Axis for AD

### 3.1. Innate Immunity and Lipid Metabolism

Alzheimer’s disease displays obvious consequences in the CNS, derived from either neurodegeneration or Aβ-mediated impairment of the innate immune activity of microglia. However, Aβ synthesis was also described in anatomical areas as distant from the CNS as the intestine and liver, being found in the bloodstream as a chylomicron-like apoprotein [17] (Figure 1). Elevated low-density lipoprotein (LDL) cholesterol levels were also linked to increased risk of Alzheimer’s later in life [34]. The latter may imply direct consequences derived from genetic polymorphisms associated with cholesterol metabolism and, particularly, specific mutations in the gene encoding for ApoE. Plasma high-density lipoprotein (HDL) cholesterol levels can be increased by bacterial lipopolysaccharide (LPS), accompanied by a decrease in plasma cholesteryl ester transfer protein (CETP) concentration [35]. This could be of particular importance as HDL was recently suggested to potentially decrease the risk for AD; however, its impact on brain activity and function is not fully understood [36].

Stimulation of TLR4-activated proinflammatory responses exhibits an intricate relationship with lipid homeostasis, displaying bidirectional regulation. Activation of TLR4 stimulates lipolysis from adipose tissue or adipocytes and drives the extracellular catabolism of LDL (Low-Density Lipoprotein) aggregates [37]. Otherwise, circulating levels of fatty acids activate TLR4 signaling in adipocytes and macrophages, contributing to peripheral inflammation. Notably, taking advantage of immunometabolic strategies, it could be possible to significantly drive a selective orientation of TLR4 downstream signaling by engaging adaptor molecules, such as either MyD88 or TRIF/TICAM, to influence its inflammatory potential (Figure 1). In contrast to experimental studies, a longitudinal population-based study showed that saturated fatty acids appear to be inversely associated with the risk of AD [38]. No evidence of a protective effect of n-3 fatty acids against dementia was found [38]. Otherwise, studies on metabolic profiling of brain tissue samples of patients with varying degrees of AD pathology suggested that unsaturated fatty acid metabolism is significantly dysregulated [39]. Although TLR4 is not a receptor for saturated fatty acids, polyunsaturated fatty acids, particularly docosahexaenoic acid (DHA), were shown as inhibitory towards TLR4 [40]. In relation to TLR4 activation, recent studies support a pivotal role for SETIs, which exert potential important roles in plant physiology and animal (immune)nutrition. While SETIs can interact with TLR4, they mainly differ from each other in their capacity to engage different adaptor molecules (MyD88, TRIF/TICAM) associated with TLR4 [13,41,42]. These differences seem to rest on the transport of prosthetic groups (i.e., glucuronide, glucoside), which determine TLR4 downstream signaling via the adaptor molecule TRIF [13]. At the intestinal level, SETIs exert immunostimulatory effects that promote selective functional differentiation of the myeloid population [13,41,42], whose phenotype contributes to either aggravating or controlling inflammation in vivo (Figure 1). These effects can be reflected even at the liver level, thus affecting lipid homeostasis and fat accumulation. Moreover, the administration of these extracts to animals kept under a high-fat diet enabled modifications in gut microbiota composition [15] that could have positive effects in AD patients. Recently, a strong connection between inflammatory bowel disease and noncoding RNAs (ncRNAs) was also reported [43]. The potential use of immunonutritional vesicles (i.e., containing molecules to selectively drive TLR4-mediated responses and ncRNAs) could represent potential novel approaches for tackling aberrant CNS inflammation and microglia function [44].

### 3.2. Innate Immunity within the Gut–Liver Axis

It is worth keeping in mind the TLR4-mediated potential to influence endocrine and metabolic effects. Intestinal TLR4 were identified as a key factor determining insulin resistance [45]. In parallel, TLRs are highly expressed in hematopoietic cells, including macrophages, dendritic cells, neutrophils and lymphocytes. Thus, innate immune signals that stem from the intestinal level significantly contribute to the activation and propagation, as well as aggressiveness, of both innate and adaptive immune responses. For instance, the TLR4 homologue TLR9, which recognizes cytosine–guanosine-containing DNA oligodeoxynucleotides from microbes, parasites and viruses [46], served as an immunostimulatory tool to limit TLR4 signaling, reducing both the cortical and vascular amyloid burden in preclinical models [47]. Moreover, the close interplay between NOD1 and TLR4 [48], which acts as a key determinant influencing the up-regulation of NOD1 [49], may contribute to the (re)programming of epigenetic-based ‘trained immunity’ responses, aggravating the molecular consequences. The more proinflammatory profile appears to be dependent on a non-synergistic activation of these receptors. Previous research studied potential mechanisms for NOD1–TLR4 crosstalk, suggesting that synergy between them develops when TLR4 uses proximal adaptor molecules, such as TRIF. This enhances signaling through the NOD1 pathway by upregulating RIPK2 mRNA expression and preventing loss of RIP2 protein [48]. In this sense, there is potential for the development of so-called ‘trained immunity’ for chronic inflammatory diseases, which involves epigenetic and metabolic reprogramming of macrophages, and to date has not been tested for AD. Within the epigenetics of AD, several genes are hypermethylated (*APOE, MTHFR, MAPT, SORB3*), and others are related to the production of the Aβ peptide (*PSEN1, APP, PP2A, CREB5, S100A2, BACE*) and remain hypomethylated.

NOD1 represents a novel target for adipose inflammation in obesity, due to its important roles in immune response(s) and inflammation in adipocytes. Obesity and its associated conditions were suggested as a possible causative involved in AD [50,51]. NOD1-mediated lipolysis, among others, promotes diacylglycerol (DAG) accumulation and successive inflammation via the PKCδ–IRAK axis in adipocytes [52]. Interestingly, the activation of PKCδ stimulates IRAK1/4 and consequently increases the production of proinflammatory cytokines such as IL-1β, IL-18, IL-6, TNFα and MCP-1. However, it has been suggested that upregulating IL-6 or IL-1β, or downregulating anti-inflammatory cytokines such as IL-10 and TGF-β, could be beneficial to attenuating Aβ load as well as enhancing the phagocytosis of potentially detrimental proteins and debris [53]. Lipidomic studies demonstrated the early accumulation of DAG in the frontal cortex and plasma in patients suffering from mild cognitive impairment [8,54]. These alterations could be associated with several single nucleotide polymorphisms (SNPs) and polygenic risk scores (PRS) of the disease at different levels. The most significant associations implicated FERMT2 and MS4A6A with all lipid classes, while ABCA7 had a differential association with more than half of the diglyceride lipids (52.6%) and phosphatidylinositols (57.1%) [8]. In addition, 43.4% of the sphingomyelins class was differentially associated with CLU. This points out the key, if not causal, role that the dysregulation of lipid homeostasis could play in cognitive impairment and Alzheimer’s disease late in life. Notably, the apolipoprotein E (APOE) 4 allele is the strongest risk factor for sporadic AD exclusive of age, and APOE4 carriers do not respond to dietary supplements as well as to the DHA present in fish [55]. As an explanation, the author hypothesized that the presence of DHA in phospholipid form in fish could influence whether DHA is metabolized. Notwithstanding, the role of NOD1 in AD could be extended to the suppression of adipocyte differentiation and expression of nuclear receptors such as PPARγ, as well as CCAAT/enhancer binding protein-α, fatty acid binding protein-4, and leptin [56]. Several clinical trials already revealed promising results using PPARγ agonists [57], and leptin expression levels and signaling were positively linked to the disease as a protective factor [58], representing attractive therapeutic targets for the treatment of AD.

Particular attention was given to cyto-therapeutic interventions that utilize M2 microglial polarization as a potential option for treating neuroinflammation and AD. However, while shedding light, available data also cast a few shadows. TREM2 supports a metabolic program that maintains alternative activated M2 macrophages [59], implying an increased lipid metabolism. Here, apolipoprotein E (apoE) exerts potent anti-inflammatory effects, while signaling via VLDL-R or apoER2 promotes macrophage conversion from the pro-inflammatory M1 to the anti-inflammatory M2 phenotype [60]. In research reports over the last few years, we learned a great deal regarding altered lipid homeostasis and metabolism, which could represent an important factor in the development and/or severity of AD. The search to better control the inflammatory environment in the CNS is of importance from a pharmacological approach. The important impact of defined phospholipids, favoring a functional phenotype of macrophages that displays a more controlled proinflammatory character, is well known. Notably, the presence of ethanolamine phospholipids in the proteoliposome of AD patients was found to weaken γ-secretase activity [61]. Growing evidence suggests that not only phospholipids, but also plasmalogens, a subtype of phospholipids, have a close association with AD (for example, decreased ethanolamine plasmalogens). Plasmalogens appear to inhibit the endocytosis of TLR4, attenuating the inflammatory signal in microglial cells [62]. These biochemical alterations should also be considered in the context of those derived from Krüppel-like factors (KLFs), a family of transcription factors that can potentially worsen (KLF4, KLF14) or improve (KLF2, KLF8) the disease. KLFs play regulatory roles in glucose, lipid and amino acid metabolism, coordinating systemic metabolism in a steady state and in the face of metabolic stresses such as fasting [63]. It is worth bringing up here the relationship of TLR4 with factors such as KLF14, which can result in down-regulation via TLR4/TRIF promotion of ERK1/2 phosphorylation and thereby help to develop a synergistic action with NOD1 to underpin peripheral inflammatory responses in AD patients. Due to the intricate relationship of AD with peripheral inflammation, the bloodstream may serve as the target anatomical location to prevent and/or ameliorate inflammatory signals. A clear example is the protective role of hematopoietic NOD1 deficiency on high fat diet-induced proinflammatory macrophage activation [64].

### 3.3. Microbial Influence

Alterations in intestinal microbial ecology cause mucosal barrier impairment and increased proinflammatory signals derived from TLR4 stimulation (Figure 1). Accordingly, inflammatory bowel disease was linked to a doubling in dementia risk [65]. Hypoxia-induced signaling by hypoxia-inducible factors (HIFs) and NFκB can either promote or counteract these intestinal inflammatory responses [66]. These signals are closely associated with the epithelial cells–commensal microbiota crosstalk. This determines a dynamic exchange of gaseous signals that was shown to be critical for nutrient absorption, intestinal barrier function, and innate and adaptive immune responses. Hypoxia enhances lipogenesis, promoting fatty acid uptake, accumulation, and use, as well as LDL and VLDL through inducing VLDL-R [67]. Overall, basic and clinical investigations suggested an association between hypoxia–ApoE4–cognitive impairment [68]. However, the pathophysiologic mechanisms underlying the observed interaction among hypoxia, ApoE, and cognition remain to be clarified. The stabilization of intestinal hypoxia via microbiota-derived short chain fatty acids (SCFAs) helps to control host energy homeostasis [69]. Recent research identified macrophages as the main target of diet, exerting key regulatory roles in the control of adiposity and energy storage [70]. SCFAs such as butyrate can play several important roles influencing AD, including among others (i) adipose tissue inflammation, (ii) promoting Aβ plaque deposition, and (iii) driving selective macrophage differentiation and activity. The latter can have important consequences in vivo, since butyrate induces an antimicrobial phenotype with a shift in macrophage metabolism through histone deacetylase 3 (HDAC3) inhibition [71]. Notably, the inhibition of HDAC3 restores the Aβ oligomer-induced plasticity deficit in hippocampal CA1 pyramidal neurons [72]. Otherwise, the short-chain fatty acid receptor GPR43 for butyrate seems to play distinct functions depending on macrophage type (M1 or M2) [73]. Thus, whether hypoxia-related pathways could be proposed as potential therapeutic targets within the gut–brain axis for inflammatory processes should be further investigated and established. In addition, despite the well-established role of hypoxia in carbohydrate metabolism, its role in the regulation of lipid homeostasis otherwise remains ill defined. Moreover, the study of population-based differences in intestinal microbe-related metabolism is also largely inferential for AD.

## 4. Modifiable Proinflammatory Lipid Mediators in AD

### 4.1. Fatty Acids: Eicosanoids, Thromboxanes and Prostaglandins

The ϖ-6/ϖ-3 ratio is an important factor considered in studies focused on the effect of dietary polyunsaturated fatty acids (PUFAs) on AD development [25,26,27]. However, there is little debate regarding the importance of PUFAs in AD, since diets with high ϖ-6/ϖ-3 ratios are considered a risk factor related to elevated Aβ levels [74], oxidation and neuronal death [24], whereas increased ϖ-3 intake might result in an improvement in mental health [28,29]. One explanation for these dissimilar effects between ϖ-6 and ϖ-3 fatty acids resides in their metabolization to form bioactive lipids termed eicosanoids, which play a key role in modulation of the inflammatory response.

Arachidonic acid (AA) is metabolized by cyclooxygenase-2 (COX-2) and 5-lipoxygenase (5-LOX) enzymes to form pro-inflammatory molecules, such as prostaglandin E2 (PGE2), a COX-2 product involved in the development of AD through its EP receptors [75,76]. Here, macrophages play important roles in the sequential transformation of AA, involving the interaction of 15-LOX with 5-LOX to yield lipoxygenase interaction products (Figure 1). In addition, elevated thromboxane A2 (TxA2) level activated microglia and showed an association with high concentrations of amyloid precursor protein (APP) in the brains of 5X5AD transgenic mice [77], whereas abnormally high levels of prostacyclin I2 (PGI2) were linked to the accumulation of Aβ in brain tissues and hastened AD development [78]. The biosynthesis and activity of COX-2 eicosanoids are targeted by a wide range of anti-inflammatory drugs. Aspirin, the oldest and best-known NSAID, shows therapeutic effects regarding the prevention/treatment of AD [79]. However, the use of NSAIDs comes with notorious side effects, including cardiovascular effects [80] and changes in the production of eicosanoids [81], justifying the use of alternative therapies. COX-2 is in the spotlight of food science researchers as one of the main targets of polyphenols. In addition, it cannot be ruled out that PGE2 and TxA2, along with their contribution to tissue and peripheral inflammation, are differentially produced by liver macrophages in response to TLR4 stimulation [82] (Figure 1). These mediators restrain macrophage maturation and influence AA distribution and phospholipid profile, affecting functional differentiation and activity among macrophages.

An overwhelming number of studies indicated that different polyphenols (i.e., resveratrol, naringenin, phloretin) reduce the expression/level of COX-2 as a protective mechanism against AD [83,84]. These studies evidence a direct link between the release of PGE2 and TxA2 by TLR4-activated microglial BV2 cells. To the best of our knowledge, whether polyphenols target the COX-2 pathway through the modulation of the expression of PG receptors or EP and DP receptors, or through binding to them, remains undetermined. 5-Lipoxygenase (5-LOX) is an important enzyme directly involved in neuroinflammation through the formation of leukotrienes (LTs) [85]. The 5-LOX products LTB4 and Cys-LTs play a critical function in 5-LOX-related βA formation [86,87]. A recent systematic review summarized preclinical and human studies describing the beneficial roles of polyphenols in modulation of the 5-LOX pathway [88]. Unexpectedly, the effect of polyphenols on AD development targeting 5-LOX is an unexplored field, and the included studies overlooked this interaction as a mechanism. Overall, the modulation of 5-LOX could affect different innate immune myeloid cell developmental processes, including, among others, macrophage metabolism and specialized proresolving mediators, as well as gene transcription and protein expression in human neutrophils. In addition, 5-LOX and COX-2 are involved in the biosynthesis of 5-hydroxy-prostaglandins (5-OH-PGE2 and 5-OH-PGD2) [89] and hemiketal eicosanoids (HKE2 and HKD2) [90], which are the result of crossover between the 5-LOX and COX-2 pathways [91,92]. These metabolites are truly novel molecules and information on their biological activities is limited. 5-OH-PGs failed to activate EP and DP receptors [89], suggesting a different biological activity of PGE2 and PGD2. HKE2 and HKD2 promote inhibition of platelet aggregation [93] as well as tubulogenic promotion and migration in endothelial cells [90], which are processes related to chronic inflammatory diseases such as cardiovascular diseases and atherosclerosis. The formation of HKs in the nervous system is plausible due to COX-2 and 5-LOX expression in both neurons and glia; however, their role in AD remains unknown. Furthermore, these eicosanoids could be an interesting therapeutic target to ameliorate neuroinflammation, considering that specific inhibitors of 5-LOX and COX-2 [94] and polyphenols, including urolithins and curcumin [94,95], inhibit the biosynthesis of these molecules.

Eicosanoids derived from ϖ-3 fatty acids (i.e., EPA and DHA) have remarkable pro-resolution and(or) anti-inflammatory effects compared to AA-derived pro-inflammatory eicosanoids [96]. EPA is the precursor of the E-series resolvins (RvE1), while DHA is metabolized to produce D-series resolvins (RvD1 and RvD2), protectins (PD1) and maresins (MaR1) through the activities of Cytochrome P450, COX-2, 5-LOX, 15-LOX and 12-LOX [97]. The levels of these molecules are lower in the entorhinal cortex of AD patients than in age-matched volunteers, which might benefit AD development considering their stimulatory effect on microglia cells to uptake the pro-inflammatory Aβ42 [98]. Modulation of anti-inflammatory/pro-resolution lipid mediators’ biosynthesis through supplementation of ϖ-3 fatty acids is an attractive strategy, widely approached against AD. In this regard, the analysis of blood samples from healthy volunteers showed the presence of a wide range of lipid mediators, such as RvD1 and RvD2, in plasma [99] and significantly higher levels of RvE1, 14(R,S)-, 17(R,S)- and 18(R,S)-hydroxyeicosatetraenoic (HETE) acids. The consumption of drinks enriched with ϖ-3 fatty acids and antioxidants (pomegranate and chokeberry) increased RvD1 biosynthesis and Aβ phagocytosis in macrophages from AD patients [100]. However, the relation between ϖ-3 fatty acid consumption and beneficial effects against AD is unclear, considering the negligible results observed (i.e., improving dementia or cognitive impairment) in clinical trials [101,102].

### 4.2. Inflammatory Regulation

The consumption of plant-derived food is associated with amelioration/improvement in AD, as well as other chronic inflammatory diseases. Pomegranate is a widely consumed fruit recognized as a beneficial foodstuff for the nervous system. Enhanced brain activation, increased memory retention and neurophysiological improvement are positive effects associated with the consumption of pomegranate described in clinical trials [103]. Soy is another interesting source of compounds that could be effective for cognitive function improvement in aged adults and AD patients [104,105,106]. Ellagitannins (ETs) and isoflavones, present in both pomegranate and soy (among others), might be (partially) responsible for these health benefits. A common detail regarding ETs and isoflavones is that they are suitable substrates for the gut microbiota to form microbial derived metabolites. Ellagitannins and ellagic acid undergo gut microbiota metabolism to yield a group of microbial-derived metabolites termed urolithins (Uro-A, Uro-B, IsoUro-A and Uro-C among others). Similarly, isoflavones such as daidzin and genistin (glycosides) and daidzein and genistein (aglycones) reach the gastrointestinal tract in their original form, where they interact with gut microbiota, resulting in the formation of equol and(or) O-desmethylangolesin (ODMA) [107]. This different metabolism sets a basis for differentiating diverse population groups based on their capacity to produce (or not) microbial metabolites: (i) urolithin ‘metabotypes’ (UM): UM-A (Uro-A producers as final urolithin), UM-B (specific production of IsoUro-A and Uro-B, in addition to Uro-A), and UM-0 (urolithin non-producers); (ii) ODMA- and equol-producer phenotypes (metabotypes) [107,108].

Studies focused on the use of natural compounds to ameliorate the neuroinflammation associated with AD development encompass a wider spectrum of polyphenols than only urolithins or equol. One of the anti-inflammatory effects investigated is related to the polarization of macrophages favoring the M2 anti-inflammatory phenotype. Well-known polyphenols such as quercetin and curcumin activate M2-type polarization in vitro and in vivo, exerting a protective function against neuroinflammation [109,110]. In this regard, genistein also promotes M2 polarization by targeting the TLR4 signaling pathway [111]. In addition, quercetin, curcumin and resveratrol are molecules with the capacity to protect BV2 cells from oxygen/glucose deprivation and LPS-induced inflammation by targeting TLR4/NF-κB signaling [112,113,114]. The free forms of urolithins, including Uro-A and Uro-B [115,116] and curcumin [117] were shown to be effective at inhibiting NF-kB activation and related downstream pathways (ERK, p38, JNK and Akt) in BV2 microglia cells. This inhibitory effect could explain, at least in part, one of the mechanisms of the modulation of the biosynthesis of anti-(such as IL-10) and pro-inflammatory cytokines (TNF-α, IL-6, IL-1β [115,116,118], the down-regulation of NLRP3 gene expression [117], and the lower level (mRNA and protein) of cyclooxygenase-2 (COX-2) [108,119,120] in microglia cells treated with polyphenols. A recurrent pitfall in the design of these in vitro studies is the overlooking of the bioavailability of phenolic compounds. A common practice is to expose systemic cells to physiologically irrelevant polyphenols (i.e., those that do not reach systemic tissues) using concentrations higher than those detected in vivo, limiting the relevance of the results reported [121]. Notably, there is less information regarding the biological activity of phase-II metabolites, which are the major circulating molecules. Evaluation of neuroprotection in H_2_O_2_-treated neuroblastoma SH-SY5Y cells in the presence of Uro-A, Uro-B and IsoUro-A or their conjugated (glucuronides and sulphates) metabolites showed a reduction in induced cytotoxicity, although the conjugated metabolites were less active [122]. Attenuation of insulin resistance, gut microbiota dysbiosis, cognitive impairment and neuroinflammation were effects observed in Aβ_1–42_-injected C57BL/6J mice fed diets containing quercetin 3-glucuronide (50 mg/kg b.w.). Analysis of the effect in hippocampus tissues revealed inhibition of Tau phosphorylation, modulation of cytokine biosynthesis (TNF-α, IL-1β, IL-6, INF-β, IL-5, IL-10) and amelioration of the effects of Aβ_1–42_ on significant signaling pathways, including MAPK, Akt, CREB and IRS [123].

Animal studies support the hypothesis that the microbial metabolite Uro-A is the major bioactive compound formed after pomegranate consumption. Amelioration of memory deficits, reduction in cognitive impairment and improvement in spatial memory are common effects observed in AD-induced mouse models that consumed Uro-A-enriched diets [108]. The molecular mechanisms associated with these benefits include reduction in Aβ-plaque deposition and tau phosphorylation, APP down-regulation, autophagy induction, and attenuation of glial cell activation and inflammation (lower levels of pro-inflammatory cytokines and inhibition of NF-κB pathway activation) [108,124,125,126]. Equol is in the spotlight as a possible protective factor against cognitive impairment [127,128]. The therapeutic benefits of equol in the nervous system involve the potentiation of brain metabolic activity targeting, in vitro and in vivo, the mitochondria of neuronal cultures. The mechanisms associated include modulation (expression, post-transcriptional modifications and activity) of mitochondrial bioenergetic enzyme activity (including pyruvate deshydrogenase, α-ketoglutarate dehydrogenase, complex I—NADH dehydrogenase and complex IV—cytochrome c oxidase), reduction in lipid oxidation, and up- or down-regulation of related gene expression [129]. Exploration of further mechanisms implies estrogen receptor-α (ERα) as a receptor through which equol might exert its effects. A recent study described 1 µM equol as equally efficient as 17β-estradiol in reducing cytoxicity, avoiding cell cycle re-rentry, lowering cyclin D1 expression and ERK1/2 activation, and increasing ERα and steroid receptor coactivator-1 (SRC-1) level in Aβ_25–35_-treated SH-SY5Y cells [130]. These results highlight the importance of the gut microbiota as a key element regarding the neuroprotective effect of ETs and isoflavones. Microbial dysbiosis could be responsible, at least in part, for physiological variations in responses to AD treatment. However, studies of population-based differences remain overlooked. In this sense, it is worth bringing up here the recent utilization of lipid-based nanosystems to deliver polyphenol-related compounds, improving bioavailablity [131].

## 5. The Innate Immune Reflex of Metabolic Interventions in Innate Immunity in AD

Pharmacological approaches to the unmet need for treatment modalities with increased effectiveness in AD remain unresolved. This has promoted intense research to address the modulation of innate immunity by developing different molecules that can stimulate immune cells [6]. However, despite the evidence indicating influence by exogenous and endogenous factors, such as diet, nutrients, environmental exposure, and microbiome among others, on interactions between neuro- and peripheral inflammation, integrative analyses of these factors and pharmacological approaches lag. Non-pharmacological approaches, aiming to target key immunometabolic processes through the production of specific lipid mediators, received increasing attention due to their potential to slow down the progression of AD. Immunometabolic interventions able to act at the level of peripheral inflammation and production of proinflammatory mediators, as well as to bias the selective functional differentiation of monocytes towards a specific phenotype and activity, would have the potential to overcome this transdisciplinary gap for precision medicine.

Bioactive immunometabolic nutrients and diets influence, by various biological means, the activation or blocking of signaling pathways at the cellular level: metabolic control through energy metabolism, ROS metabolism and lipid metabolism; epigenetic modification through DNA methylation, histone modification and non-coding RNA regulation; and immunological regulation through microglial activation (CX3CL1 inhibition) and astrocyte activation (TLR4) [132]. To date, no evidence was provided demonstrating potential protective effects of individual foods on memory, cognitive decline and AD, except for red wine, which only reduced the risk of AD in men, while increasing it in women [133]. The dietary patterns studied concerning AD were focused mainly on the metabolism of lipids to mediate the disease’s prototypical neuroinflammation. Glucose metabolism and insulin signaling were also studied, demonstrating a direct link between AD and Type II Diabetes (T2D). Specifically, the risk of developing dementia doubles in the case of patients with diabetes. A decrease in the number of insulin receptors in the brains of elderly patients with Alzheimer’s disease along with a decrease in mRNA expression were observed, producing deviations in the insulin receptor–IRS1–AKT–mTOR signal pathway and its various serine kinases, giving rise to amyloid-β and tau protein lesions [6]. Insulin transport by capillary endothelial cells at the BBB proved to be affected by many systemic factors, including those related to metabolic features occurring in obesity [134]. Obesity is also a significant risk factor for AD, and the close relationship between obesity and T2D is known. A high-fat diet intake, associated with excessive body weight gain and the development of a prediabetic state, was also associated with hippocampal neurogenesis and cognitive impairment in murine models [135]. Otherwise, the ketogenic diet, a high-fat, protein-controlled, low-carbohydrate diet, was postulated as a possible nutritional intervention to manage neurological diseases. The ketogenic diet may contribute (i) to a reduction in amyloid and tau protein conglomerates, (ii) to increases in the activity of ATP-sensitive potassium channels, (iii) to increases in BDNF protein in cortical neurons, (iv) to protection against neuroinflammation by decreasing microglial activation and reducing the expression of proinflammatory cytokines, and (v) to enhancement of free radical scavenging and activity in antioxidant systems. Currently, this diet is considered the standard non-pharmacological treatment of epilepsy and is under investigation in patients with AD [136]. To the best of our knowledge, five clinical trials related to AD were conducted to date (2018-present). The major conclusions of these studies indicate that the ketogenic diet normalizes carbohydrate metabolism in the brain, reduces insulin levels, increases insulin sensitivity, and improves cognitive performance [137,138,139,140,141]. These represent major nutrient-associated conditions influencing monocyte-derived macrophage polarization towards an inflammatory phenotype, as well as microglia polarization.

## 6. Conclusions and Future Perspectives

While non-pharmacological strategies gained acceptance in the targeting of peripheral proinflammatory processes affecting neuroinflammation in AD treatment, many immunometabolic bioactive compounds remain biomedically ill-defined for clinical use. The quality of the bioactive compound depends not only on genetic factors in the natural source, but also on extrinsic ones such as environmental agronomic conditions and technological practices for its preparation. Altogether, only in completion with their dynamics within the gastrointestinal tract can the functional features and bioactivities of the compounds be determined. To improve the bioavailability of immunometabolic neuroprotective compounds and thereby their effectiveness, making them useful in clinical practice, biocompatible vesicles as well as nanocarrier-based strategies could be used [44,131]. A better understanding of the interaction of those vehicles with human myeloid cells—including monocytes and macrophages derived from those—can help significantly improve phagocytic activity and functional polarization stimuli, favoring an anti-inflammatory peripheral profile of monocytes which can influence that of the microglia (Figure 1).

Although several of the abovementioned non-pharmacological strategies and im-munometabolically active compounds were evaluated in various stages of preclinical trials, improved research into immunometabolic bioactive natural compounds within the life sciences is needed, along with large-scale studies. An integrative perspective addressing this gap will contribute to the transdisciplinary field of molecular pathological epidemiology, offering research frameworks to integrate the modulation of peripheral innate immunity and link both genetic and environmental exposure to cognitive impairment and pharmacological treatments for ‘specific’ patients based on patient and AD characteristics. Altogether, this could have important consequences in enhancing remarkable therapeutic effects in AD patients, thus improving the health and quality of life of patients.

## Data Availability

Not applicable.
